# An Evolution Based Biosensor Receptor DNA Sequence Generation Algorithm

**DOI:** 10.3390/s100100330

**Published:** 2009-12-31

**Authors:** Eungyeong Kim, Malrey Lee, Thomas M. Gatton, Jaewan Lee, Yupeng Zang

**Affiliations:** 1 Advanced Graduate Education Center for Electronics of Jeonbuk and Information Technology-BK21, Jeonju, Jeonbuk, 561-756, Korea; E-Mail: rotnrwk@kongju.ac.kr; 2 The Research Center of Industrial Technology, School of Electronics & Information Engineering, ChonBuk National University, 664-14, 1Ga, DeokJin-Dong, JeonJu, ChonBuk, 561-756, Korea; 3 The School of Engineering and Technology, National University, 11255 North Torrey Pines Road, La Jolla, CA 92037, USA; E-Mail: tgatton@nu.edu; 4 School of Electronics and Information Engineering, Kunsan National University, San 68, Miryoung-dong, Gunsan, Jeollabuk-do, 573-701, Korea

**Keywords:** biosensor, DNA computing, DNA sequence, TSP (Traveling Salesman Problem), evolution programming

## Abstract

A biosensor is composed of a bioreceptor, an associated recognition molecule, and a signal transducer that can selectively detect target substances for analysis. DNA based biosensors utilize receptor molecules that allow hybridization with the target analyte. However, most DNA biosensor research uses oligonucleotides as the target analytes and does not address the potential problems of real samples. The identification of recognition molecules suitable for real target analyte samples is an important step towards further development of DNA biosensors. This study examines the characteristics of DNA used as bioreceptors and proposes a hybrid evolution-based DNA sequence generating algorithm, based on DNA computing, to identify suitable DNA bioreceptor recognition molecules for stable hybridization with real target substances. The Traveling Salesman Problem (TSP) approach is applied in the proposed algorithm to evaluate the safety and fitness of the generated DNA sequences. This approach improves efficiency and stability for enhanced and variable-length DNA sequence generation and allows extension to generation of variable-length DNA sequences with diverse receptor recognition requirements.

## 1. Introduction

A biosensor, which is composed of a bioreceptor and a signal transducer, is a device for selectively detecting specific substances [1,2]. Biosensor design must consider how to select the target substance, how to initiate the selective hybridization with the target substance using a simple signal system, and how to control the mechanism for completing the hybridization operation and establish communication of the substance information [3]. DNA based biosensors utilize receptor molecules that allow hybridization with the target analyte. However, most DNA biosensor research uses oligonucleotides as the target analytes and does not address the potential problems of real samples. The identification of recognition molecules suitable for real target analyte samples is an important step towards further development of DNA biosensors [4]. DNA based bioreceptors perform hybridization, similar to those found in DNA computing operations [5–7]. DNA computing was proposed by Adleman in 1994 and was demonstrated using the Hamiltonian Path Problem (HPP) approach [8]. The process of DNA computing, sequence design and recognition DNA sequence generation algorithms has been documented and the implementation of a DNA chip is in progress [9–12]. The approach used in the identification of suitable DNA sequences for DNA computing operations is applicable to the identification of DNA receptors for molecule recognition in DNA biosensors.

This study analyzes the problems and current solutions in identifying suitable DNA material as a recognition molecule in DNA computing. A new algorithm for identifying DNA molecule recognition bioreceptor sequences that integrates evolution programming and TSP is introduced, developed and evaluated, and the conclusions are presented. Section 2 presents a brief overview of the background and current state-of-the-art in DNA computing. Section 3 explains the problems and deficiencies of the existing approaches and introduces the proposed algorithm. Section 4 provides an evaluation of the safety and efficiency of the generated bioreceptor recognition molecule DNA sequences. Section 5 contains an interpretation of the evaluation of the proposed algorithm and the conclusions and recommendations for further research.

## 2. DNA Computing

DNA computing is a biologically based computer technology that uses chemically synthesized DNA as a means of computation and a medium of information storage. A double helix DNA strand is composed of the four bases of A (Adenine), T (Thymine), C (Cytosine), and G (Guanine). These bases have a memory function that can save large quantities of data. A and G are in a complementary Watson-Crick bond with T and C, respectively [13–15]. The complicated base pattern mixture contains a piece of hereditary information and is read by an enzyme that naturally occurs in the human body. In addition, enzymes, together with biological experiment methods, are being used as operators for DNA computing. Representative operators are melting, annealing, ligation, PCR and Gel electrophoresis. Table 1 shows a comparison of the characteristics between DNA and silicon based computers [16].

The basic DNA computing model formulated by Adleman in 1994 solved the Hamiltonian Path Problem (HPP), which is a combinatorial optimization problem, through a biological experiment. In solving the HPP, the process expressed possible solutions in DNA codes to find the path that includes all vertexes from the start vertex to the end vertex, exactly once. It then produced candidate solutions among sequences bound through synthesis and separation, and determined if there was an acceptable solution. [8]. Adleman’s algorithm for generation of suitable DNA sequences is modified in the proposed algorithm to produce suitable DNA sequences for recognition molecules in DNA biosensors, as presented in the next section.

## 3. DNA Biosensor Recognition Molecule Receptor DNA Sequence Generating Algorithm

Recent research reports a biosensor using DNA as the bioreceptor and examined the possibility of a recognition molecule bioreceptor using fixed-length DNA sequences and an analysis of images from biochemical experiments [17]. A fixed-length DNA sequence cannot reflect the characteristics of DNA accurately. Therefore, it is difficult to secure stability in consideration of the diverse properties of DNA encountered during experimentation. In addition, if an enzyme is used in signal transduction, fixed-length DNA sequences may produce unexpected results.

**Figure 1.**
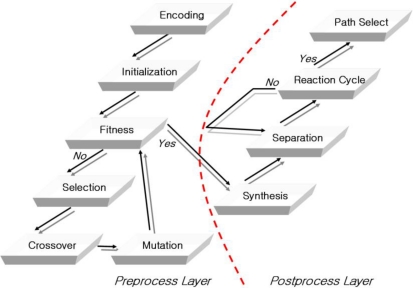
The flow of the recognition molecule receptor DNA sequence generation algorithm.

To solve these problems, this paper proposes a recognition molecule DNA sequence generation algorithm that reflects the properties of DNA and allows stable hybridization, when DNA is used for molecule recognition in the bioreceptor. The proposed bioreceptor recognition molecule DNA sequence generation algorithm applies an evolution algorithm for the generation of the initial recognition molecule DNA sequences. This allows more stable expression of the DNA than existing fixed-length receptor DNA sequence generation, and accurately reflects the characteristics of the DNA. As shown in Figure 1, the structure of the recognition molecule DNA sequence algorithm is an enhancement of Adleman’s DNA computing algorithm. It is comprised of a pre and post-process and takes into account the characteristics and capabilities of using TSP in the approach.

**Figure 2.**
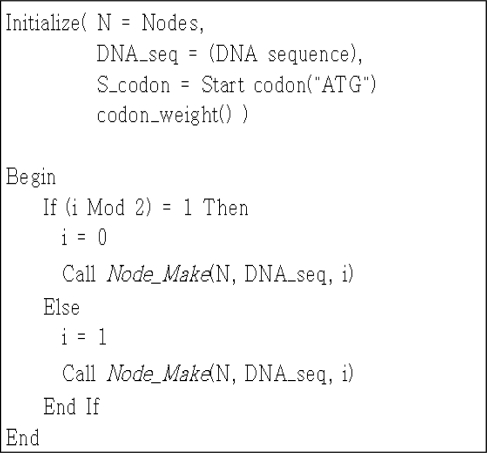

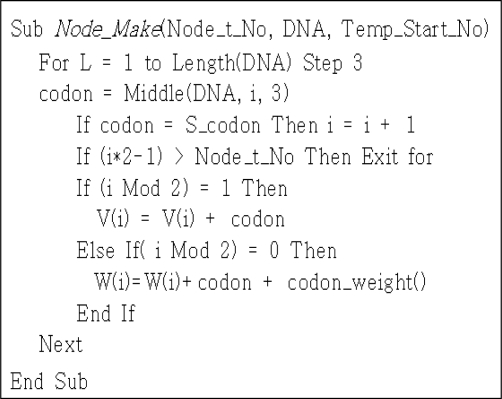
Procedure to express vertexes and weights.

First, the preprocess layer is divided into the encoding, initialization and fitness evaluation methods. The encoding method generates variable-length edges, including vertexes and weights, using the evolution algorithm, in order for the given sequence to reflect the characteristics of DNA molecules. The vertexes and edges cannot be expressed directly, and they are converted to DNA sequences using the procedure illustrated in Figure 2. First, the position of start codon (ATG) is identified, and DNA code from the (*i*)th start codon position to the codon in front of the (*i* + 1)th start codon position is expressed as a vertex. Then, DNA code from the (*i* + 1)th start codon position to the codon in front of the (*i* + 2)th start codon position is expressed as a weight. However, if the DNA code does not begin with a start codon, the vertex from the beginning of the DNA code to the codon in front of the ith start codon position is used.

**Figure 3.**
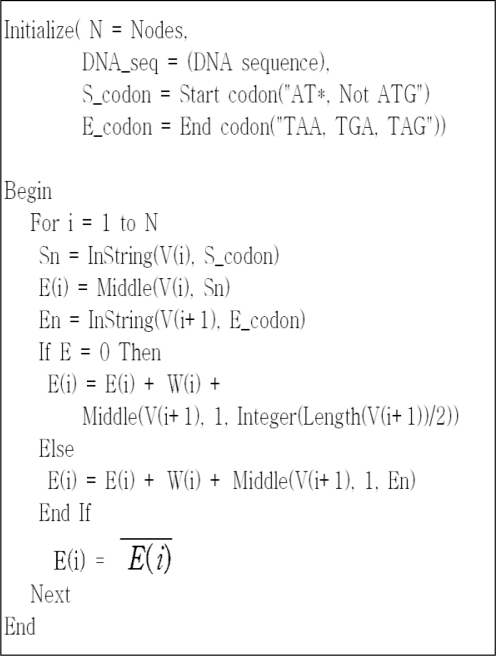
Procedure to express edges.

Edges that link the expressed vertexes follow the procedure illustrated in Figure 3 for all DNA codes. First, designate AT*(ATT, ATC, ATA), which appears first in vertex Vi, as E_(i)_ and stop codons TAA, TGA and TAG, which appear first in V_(*i*+1)_ as E_(*i*+1)_. Then, encode an edge between the two ver-texes. If there is no stop codon, then take the DNA code of 1/2bp (base pair) of V_(i+1)_ as the edge.

**Figure 4.**
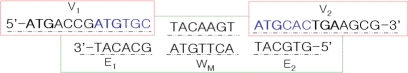
An example of path creation containing a weight.

Through the procedures in Figures 2 and 3, DNA sequences with vertexes, weights and edges are generated, and the edges containing generated vertexes and weights are integrated into a two-strand DNA sequence. After the weight sequences are included in the edges they receive complementary matching and a path to the bioreceptor DNA sequences can be generated, as in Figure 4.

Equation (1) obtains the weight of an edge using the value of the hydrogen bond conversion function for edge *i* (*N_ei_*), the actual weight of edge i (*W_ei_*), the sum of weights in the entire graph (*S_w_*), the sum of hydrogen bonds of all edges (*S_v_*) and a threshold (*θ*) determined through experimentation. An edge containing a weight is generated by including the number of hydrogen bonds for the pair of A/T’s and for the pair of G/C’s in the edge with a low and high weight, respectively:

(1) $$F_{i}=\left\{\begin{array}{c} \left|\sqrt{\frac{{Ne}_{i}}{Sv}-\frac{{We}_{i}}{Sw}}\right|\;\;\;\;\;\;\mathrm{if}\left|\sqrt{\frac{{Ne}_{i}}{Sv}-\frac{{We}_{i}}{Sw}}\geq \theta \right|\\ \mathrm{otherwise}\;\mathrm{is}\;0 \end{array}\right\}$$

Using a weight conversion equation, the length of the DNA code is adjusted with the encoded weights. This significantly expands the scope of the encoded weights and makes it possible to encode a wide range of weights with short codes.

After the encoding is completed, all sequences are removed, except for the DNA codes reflecting the selected bioreceptor requirements. In addition, a fitness evaluation, the last step of the preprocess layer, is performed by random selection to evaluate the proportion determined by Equation 2. This equation is an inverse function that applies the amino acid codes shown in Table 2. Conditions which may cause errors in biological experiments, such as inaccurate synthesis or shifting of the synthesis position, are removed in advance. If the fitness is not satisfactory, DNA codes with the highest fitness are selected and processed with a two-point crossover, which occurs only on the sequences of vertexes. Crossover points are then selected at random. For mutation, arbitrary base pairs are selected from the sequences of vertexes and mutated. This process is then repeated the same number of times as the number of generations.

(2) $$f\left(x\right)=\bar{\;x+k|\;\text{sin}\;\left(32x\right)|\;},\;\;\;\left(0\leq x<\pi ,k:\;\mathrm{Amino}\;\mathrm{acid}\;\mathrm{constant}\right)$$

The postprocessor layer performs the synthesis and separation of superior codes obtained through fitness evaluation as many times as the given number of reactions. In the process of separation, those codes that are unlikely to be solutions are removed in advance using biological operations, such as antibody affinity reaction, polymerase chain reaction (PCR) and gel electrophoresis. Lastly, the sequences in specific parts of the code are amplified by reapplying PCR. Then, a particular length of DNA sequence is abstracted with gel electrophoresis, and the path that passes through all vertexes on the graph only once is selected as the final solution, using antibody affinity.

## 4. Experiment and Evaluation

**Figure 5.**
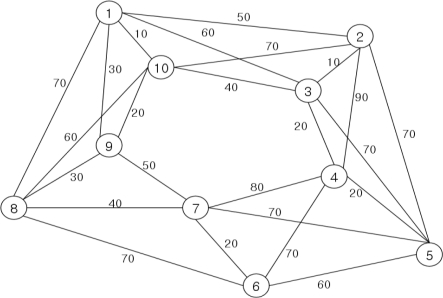
Sample TSP graph.

Testing and evaluation of the proposed algorithm compares DNA sequences that are generated through the recognition molecule DNA sequence generation algorithm, with those generated using Adleman’s DNA computing algorithm. This is accomplished by applying the TSP algorithm, as illustrated in the sample graph of Figure 5, for evaluation of the fitness of the sequences generated by each algorithm.

Simulation was implemented in C on a 2 GHz P4 PC with 512 M RAM. Because the proposed recognition molecule DNA sequence generating algorithm can perform synthesis and separation repeatedly, the number of repetitions was set at 10, and the number of reactions at 100. Accordingly, the total number of reactions was set at 1,000 (10 × 100). However, because Adleman’s DNA computing algorithm can perform synthesis and separation just once, the number of repetitions is set to 1 and the number of reactions at 1,000. This makes the same number of total reactions, as shown in Table 3. Although the length of the DNA sequences was variable in the proposed recognition molecule DNA sequence generating algorithm, the length of DNA sequences in the experiment is fixed between the ranges of 10 bp to 20 bp. This is due to the fact that Adleman’s DNA computing algorithm uses fixed-length sequences,.

As shown in Table 4, the mean fitness, the mean number of searches, and search times are measured for each algorithm. According to the results, the recognition molecule DNA sequence generating algorithm shows a higher mean fitness than Adleman’s DNA computing algorithm. Also, when the DNA codes of ACO, in which the length of vertexes was over 20 bp, are compared to Adleman’s algorithm, in which the length was 20 bp, the path search time is reduced by approximately 50 percent.

**Figure 6.**
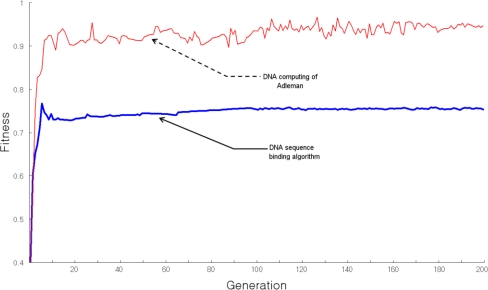
Generation Fitness.

Figure 6 shows the fitness of the generated recognition molecule DNA sequences hybridization, when evaluated using the TSP approach. The *x* axis is the number of generations and the *y* axis represents fitness. When the number of vertexes is 10, the DNA sequence generating algorithm shows uniform fitness from the 8^th^ generation, indicating stable production of recognition molecule DNA sequences. However, Adleman’s DNA computing algorithm shows irregular fitness and production of unstable bioreceptor DNA sequences. This indicates that the recognition molecule DNA sequence generating algorithm performs stable bioreceptor DNA sequence generation for variable-length states and produces the desired sequences of bioreceptor DNA codes.

**Figure 7.**
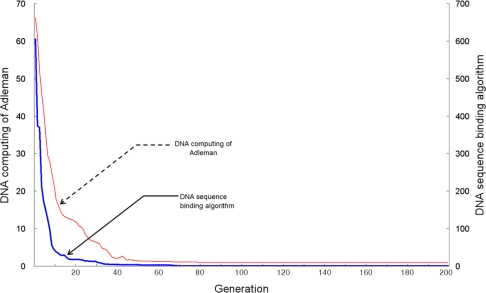
Search number for ten vertexes.

Figure 7 shows the number of searches for recognition bioreceptor molecule DNA sequences identified by the TSP fitness evaluation, when the number of vertexes is 10. It also shows that the DNA sequence generating algorithm and Adleman’s DNA computing algorithm search successful paths from the 31st generation and 52nd generation, respectively. This suggests that the proposed DNA sequence generating algorithm can find the desired bioreceptor DNA sequences within a shorter period of time, and more efficiently removes inadequate recognition molecule bioreceptor DNA sequences.

In addition, Table 5 and 6 shows DNA sequences used in this experiment. Table 5 shows variable-length vertex codes and weight codes of the DNA sequence generating algorithm, and Table 6 shows DNA codes for Adleman’s fixed-length vertex codes 10 bp and 20 bp.

In Figure 5, the optimal path used in this experiment is (V_1_, W_1→2_) → (V_2_, W_2→3_) → (V_3_, W_3→4_) → (V_4_, W_4→5_) → (V_5_, W_5→6_) → (V_6_, W_6→7_) → (V_7_, W_7→8_) → (V_8_, W_8→9_) → (V_9_, W_9→10_) → (V_10_, W_10→1_) → (V_1_), and according to the results of applying the DNA sequence bioreceptor algorithm, the optimal sequence code is as follows:

(ATGTAGC**ATT**CCCTAGG, TACGGTAGTATCAGTATGAT) →
(ATGGC**ATC**CGGG, TACATTAATAA) →
(ATGTACTCC**ATC**GT, TACGTCGCGC) →
(ATGTAGC**ATC**GTTTGGG, TACGTCGCGC) →
(ATGCTAGCTTA**ATG**AGT, TACCGCGCGCGGCCC) →
(ATGCTAACGG**ATC**TCCCG, TACGTCGCGC) →
(ATGCCT**ATA**CTTTCC, TACGCGAGGTC) →
(ATCCG**ATA**GCC, TACAATAATTATAGA) →
(ATGTTAGG**ATT**TAAG, TACGTCGCGC) →
(ATGTGG**ATC**AGC, TACATTAATAA) →
(ATGTAGC**ATT**CCCTAGG)

## 5. Conclusions and Recommendations

A bio-sensor is a chemical sensor that must select a suitable target substance, acquire and store information from the substance and convert that information into an electric signal. The molecule recognition part of the bioreceptor part must have *in vivo* affinity for the target analyte, and the transduction function part has an electro-chemical device and a transducer. This study analyzed problems in the recognition molecule portion of a bioreceptor in a DNA biosensor and proposed a recognition molecule DNA sequence generating algorithm as a solution.

The proposed bioreceptor DNA sequence algorithm used the evolution algorithm in order to reflect the properties of DNA and efficiently generated stable bioreceptor DNA structures. In addition, because it can bind variable-length DNA sequences, the algorithm can be extended to different bio-sensor requirements. A TSP algorithm was applied to evaluate the DNA sequences generated by the proposed algorithm. The results of the experiment indicated that the proposed DNA sequence generation algorithm using variable length produced higher fitness sequences, and performed searching up to 3 times faster than Adleman’s algorithm, when only using fixed lengths. These results suggest that the proposed algorithm is superior to existing methods for better molecule recognition bioreceptor DNA sequence generation and performs more efficient searching.

It is recommended that further research should be directed towards studying the composition of electro-chemical devices and transducers for converting the results of the selection function part into electric signals. This research would contribute towards furthering the practical application and realization of utilizing DNA technologies in biosensor devices. Further, it is recommended that alternative agent-based algorithms be investigated for improved efficiency over the TSP approach.

## Figures and Tables

**Table 1.** Comparison of DNA and Silicon based computer characteristics.

	**DNA computer**	**Silicon computer**
Processing	Ballistic	Hardwired
Medium	Liquid (wet) or Gaseous (dry)	Solid (dry)
Communication	3D collision	2D switching
Configuration	Amorphous (asynchronous)	Fixed (synchronous)
Parallelism	Massively parallel	Sequential
Speed	Fast (millisec)	Ultra-fast (nanosec)
Reliability	Low	High
Density	Ultrahigh	Very high
Reproducibility	Probabilistic	Deterministic

**Table 2.** Amino acid code.

Phe	16	Pro	3	His	15	Glu	13
Leu	7	Thr	5	Gln	11	Cys	6
Ile	8	Ala	1	Asn	9	Trp	19
Met	14	Tyr	18	Lys	12	Arg	17
Ser	2	Val	4	Asp	10	Gly	0
Phe	16	Pro	3	His	15	Glu	13
Leu	7	Thr	5	Gln	11	Cys	6
Ile	8	Ala	1	Asn	9	Trp	19

**Table 3.** Parameters.

**Parameter**		**DNA sequence bioreceptor algorithm**	**Adleman’s DNA computing algorithm**
population size		1,000	1,000
generation		200	200
crossover rate		0.5	0.5
mutation rate		0.01	0.01
threshold		0.3	0.3
total	max recycle	10	1
reaction cycle	reaction cycle	100	1,000
error rate in biology experiment		0.01	0.01

**Table 4.** Performance of DNA sequence bioreceptor algorithm.

**Content**		**DNA sequence bioreceptor algorithm**	**Adleman’s DNA computing algorithm**
Average fitness Values	Vertexes #10	0.747	0.927
Average Search Number	Vertexes #10	24.3	7.41
Search time(s)	Vertexes #10	3.92 × 10^4^	7.83 × 10^4^

**Table 5.** DNA code for DNA sequence generating algorithm vertexes.

**DNA sequence bioreceptor algorithm vertexes DNA code**			

**vertexes**		**weights**	
1	ATGTAGC**ATT**CCCTAGG	10	ATGTAATTATT
2	ATGGC**ATC**CGGG	20	ATGCAGCGCG
3	ATGTACTCC**ATC**GT	30	ATGTTATTAATATCT
4	ATGTAGC**ATC**GTTTGGG	40	ATGCGCTCCAG
5	ATGCTAGCTTA**ATG**AGT	50	ATGCCATCATAGTCATACTA
6	ATGCTAACGG**ATC**TCCCG	60	ATGGCGCGCGCCGGG
7	ATGCCT**ATA**CTTTCC	70	ATGCGGGCCGGCCGCGC
8	ATCCG**ATA**GCC		
9	ATGTTAGG**ATT**TAAG		
10	ATGTGG**ATC**AGC		

**Table 6.** DNA code for Adleman’3 vertexes.

**vertexes**	**Code length**	**Adleman’s vertexes DNA cod**
1	10 bp	TTGCTCTATA
	20 bp	AGTAATAGTGCAATACGTTC
2	10 bp	TACTCGCGGA
	20 bp	GACTGCATCTGATATAACCC
3	10 bp	GGTTAGTAAC
	20 bp	GGTGCAGCTGACCTACTGCT
4	10 bp	TACGCTGATT
	20 bp	CTGAACTCGTCGGTACGTAA
5	10 bp	TCAAGTTCTA
	20 bp	CATCTACGGGCCTCTATCTC
6	10 bp	AGTCAAGAGT
	20 bp	GTTTACTGACGAGGTCTCCC
